# Osteoporosis and risk of fracture in heart transplant patients

**DOI:** 10.3389/fendo.2023.1252966

**Published:** 2023-09-13

**Authors:** Marine Forien, Romain Coralli, Constance Verdonk, Sébastien Ottaviani, Esther Ebstein, Lucie Demaria, Elisabeth Palazzo, Richard Dorent, Philippe Dieudé

**Affiliations:** ^1^ Rheumatology Department, Départements Médico-Universitaires (DMU) Locomotion, Bichat Hospital Assistance Publiques des Hopitaux de Paris (APHP), Paris, France; ^2^ Cardiac Surgery and Transplantation Department, Institut National de la Santé Et de la Recherche Médicale (INSERM) U1148, Bichat Hospital (APHP), Paris, France

**Keywords:** heart transplant, bone mineral density, fractures, osteoporosis, vertebral fracture (VF)

## Abstract

**Introduction:**

Significant bone loss occurs after heart transplantation, predominantly in the first year, with increased risk of incident fractures. The goal of this study was to evaluate the prevalence of fragility fractures in a population of heart transplantation patients and to identify the independent risk factors for fractures.

**Methods:**

This was a prospective monocentric study that included patients with heart transplantation occurring < 10 years who were undergoing heart transplantation monitoring. All patients underwent bone mineral density evaluation by dual-energy X-ray absorptiometry and radiographies to establish the presence of vertebral fractures.

**Results:**

We included 79 patients (61 men); the mean age was 56.8 ± 10.8 years. The mean time between transplantation and inclusion was 32.3 ± 35.0 months. Incident fractures were diagnosed in 21 (27%) patients after heart transplantation. Vertebral fractures were the most frequent (30 vertebral fractures for 15 patients). Osteoporosis was confirmed in 22 (28%) patients. Mean bone mineral density at the femoral neck and total hip was lower with than without fracture (femoral neck: 0.777 ± 0.125 vs 0.892 ± 0.174 g/cm^2^, p<0.01; total hip: 0.892 ± 0.165 vs 0.748 ± 0.07 g/cm^2^, p<0.001), with a significant result on multivariate analysis. The mean time from transplantation to the first fracture was 8.0 ± 7.6 months.

**Discussion:**

Our study confirmed a high vertebral fracture risk in heart transplant patients, especially during the first year after transplantation.

## Introduction

Heart transplantation is an established therapy for end-stage heart failure, and the number of transplantations has increased during the past decades (1). Medical advances have improved the survival of these patients, but new challenges arise, such as osteoporosis and increased risk of fragility fractures.

Bone disease causes morbidity and mortality pre- and post-heart transplantation. The prevalence of osteoporosis at the time of heart transplantation ranges from 7% to 23% (2–4), with significant bone loss after transplantation (3–5). The significant bone loss after heart transplantation is predominant in the first year, with a prevalence ranging from 3% to 10% at the spine and 6% to 11% at the femoral neck (3–5) and 20% increased risk of fracture in the first year (3, 6).

Several factors could be linked to osteoporosis in patients with heart transplantation: prolonged immobilization, impaired renal function, lack of exposure to sunlight (vitamin D deficiency), the etiology of the heart disease (excessive alcohol consumption), cardiac insufficiency-related treatments, and immunosuppressive drugs, notably glucocorticoids. However, the prevalence of fragility fractures in patients with heart transplantation and the independent risk factors associated with incident fractures remain unclear (6–8).

Mortality is increased after a fragility fracture. A large study showed increased mortality risk for 5 to 10 years after a low trauma fracture. The age-adjusted standardized mortality during the first 5 years after fracture was estimated at 2.5 to 3.5 for hip fractures and 1.7 to 2.3 for vertebral fractures (9). To our knowledge, no study has evaluated mortality after fracture in heart transplant patients, but the results in a general population suggest a similar result in this specific population. Consequently, improving risk stratification for incident fractures could be of great value in that therapeutic intervention could help reduce the decline of bone loss (10, 11).

Therefore, the goal of this study was to evaluate the prevalence of incident fragility fractures after heart transplantation and identify independent risk factors.

## Materials and methods

### Study population

This was a cross-sectional monocentric study that included patients with heart transplantation < 10 years previous who were undergoing heart transplantation monitoring from January 2017 to December 2019. Exclusion criteria were age < 18 years, multiorgan transplantation and dialysis. All patients were evaluated by a senior rheumatologist. The following data were systematically collected: 1) demographic characteristics including sex, age, smoking status, excessive alcohol consumption (>20 g/day for women and >30 g/day for men), and diabetes; 2) the etiology of heart disease and treatment received after the transplant; 3) previous history of fractures and previous treatments for osteoporosis (calcium, vitamin D and osteoporotic therapies). Dietary calcium intake was evaluated with a specific questionnaire (12).

### Bone mineral density evaluation

BMD was measured by dual-energy X-ray absorptiometry (Hologic Inc., Waltham, MA) at the lumbar spine (second to fourth vertebrae and vertebral fractures were excluded for BMD evaluation) and the upper part of the left femur (total femur and femoral neck). The results were given as BMD (g/cm^2^) and T-scores (standard deviation). Sex-specific T-scores were based on female and male reference curves. The device was controlled by measuring a spine phantom at least three times per week throughout the study. All examinations were performed according to the manufacturer’s recommendations. Osteoporosis was defined for patients ≥50 and <50 years as a T-score ≤-2.5 SD and Z-score ≤-2 SD at the lumbar spine (L1-L4), femoral neck or total femur. Osteopenia was defined for patients ≥50 and <50 years old as a T-score ≤-1 SD and Z-score ≤-1 SD at the lumbar spine (L1-L4), femoral neck or total femur (13).

### Fracture evaluation

Data on previous fractures (location, date) were collected. All patients underwent radiography with anteroposterior and lateral views of the dorsal and lumbar spine to establish the presence of vertebral fractures. Vertebral fracture diagnosis was confirmed in the presence of a reduction > 20% of the height of the vertebra (14). An experienced rheumatologist analyzed the radiographs.

### Biochemical analysis

At inclusion, all patients were assessed for calcium, phosphorus, creatinine, parathyroid hormone, 25 hydroxy-vitamin D, ß C-terminal propeptides of type I procollagen (ßCTX), osteocalcin (N-Mid osteocalcin) and bone-specific alkaline phosphatase levels (B-ALP). Serum for biochemical analysis was obtained in the morning under fasting conditions in the same laboratory. Serum calcium (normally 2.20-2.55 mmol/l), phosphorus (normally 0.81-1.5 mmol/l) and creatinine (normally <84 μmol/l) were measured by standard automated techniques (Cobas Pro, Roche). Intact parathormone 1-84 (normally 15-65 ng/l) was measured by electrochemiluminescence immunoassay (Cobas Pro, Roche), 25 hydroxy-vitamin D (normally 75-200 nmol/l), ßCTX (normally <0.635 μg/l), osteocalcin (normally 10.4-45.6 μg/l) and B-ALP (normally 4.7-27.0 μg/l) were measured by immunoassay (IDS-iSYS). Vitamin D deficiency was defined as serum level of 25 hydroxy-vitamin D < 50 nmol/l, insufficiency 50 to 75 nmol/l (15).

### Ethics statement

The Institutional Review Board (No. 12-011) of Paris North Hospitals approved this study. Written informed consent was obtained from all participants in agreement with French bioethics laws.

### Statistical analysis

All data are expressed as mean ± SD and were compared by Mann Whitney test. Categorical data are described with number (%; percentages were calculated excluding missing data) and were compared by chi-square test or Fisher exact test, as appropriate. Univariate analysis (p<0.15) was performed to select potential explanatory variables that were then tested in the multivariable model (stepwise method). Odds ratios (ORs) and 95% confidence intervals (CIs) were estimated. P < 0.05 was considered statistically significant.

## Results

### Main characteristic of the study population

**Figure 1:**
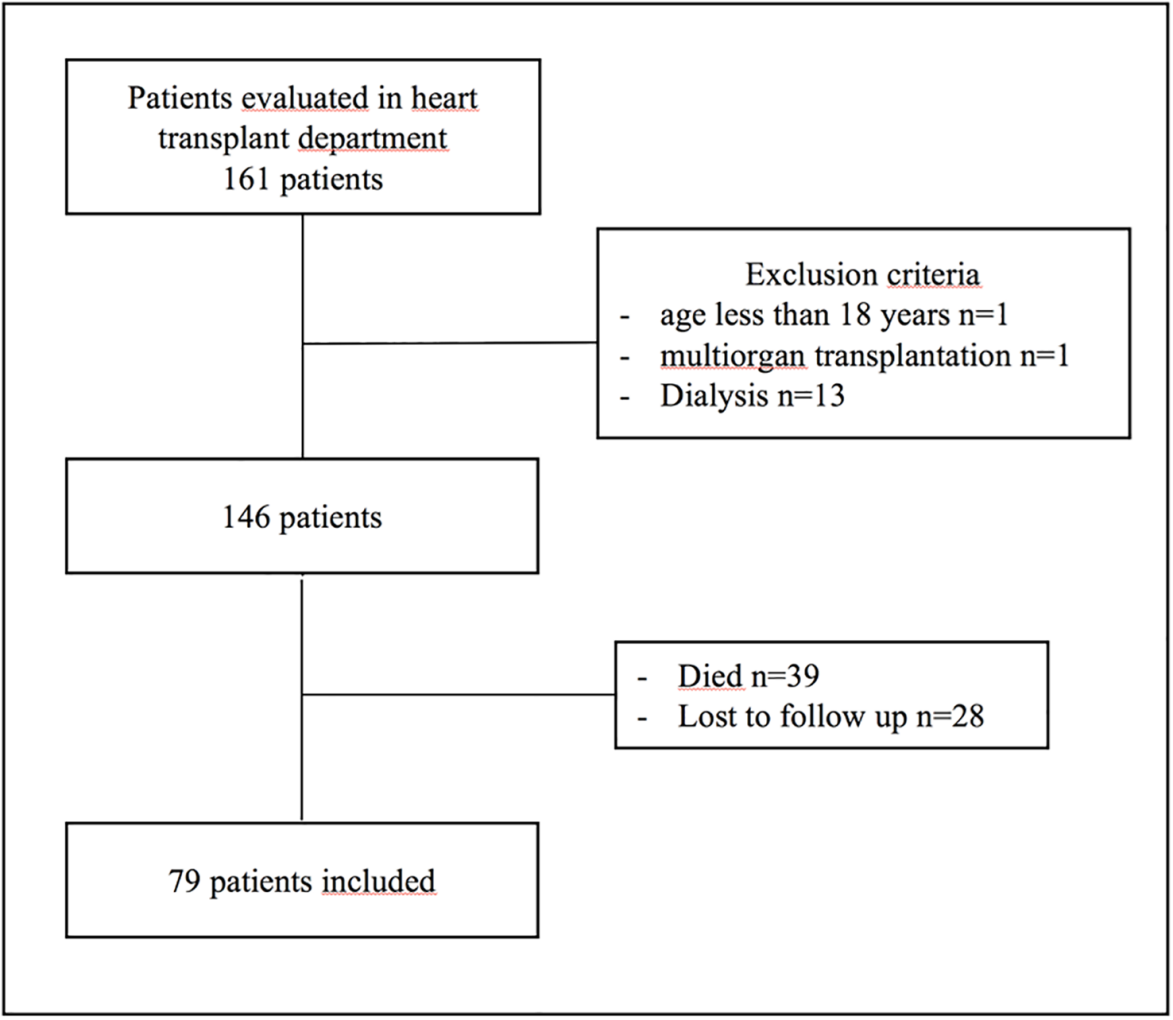
Flowchart of patients in the study.

From January 2017 to December 2019, 161 patients who underwent heart transplantation were screened for inclusion; 15 were excluded (age < 18 years, n=1; multiorgan transplantation, n=1; dialysis, n=13), 39 patients died and 28 were lost to follow-up (**Figure 1**). The baseline characteristics of the 79 patients are summarized in **Table 1**. Briefly, 61 were male (77.2%), the mean age was 54.1 ± 10.8 years, the mean body mass index (BMI) was 27.2 ± 4.99 kg/m², 49 (62%) were ever-smokers and 7 (8.9%) were current smokers. Twelve (15.2%) had excessive alcohol consumption and 29 (37%) had diabetes mellitus. Among 18 women, 15 had been menopausal for 5.3 years. The heart disease leading to transplantation was dilated cardiomyopathy for 34 (43%) patients, ischemic for 31 (39.2%), valvular cardiopathy for 6 (7.6%) and idiopathic or other cardiopathies for 8 (10.1%). The mean time from heart transplantation to inclusion was 32.3 ± 35.0 months. All patients except 2 received corticosteroids, with a mean dose of 10 ± 4.21 mg per day of prednisone. All patients received vitamin D supplementation. A total of 59 (75%) patients received ciclosporin, 67 (86%) mycophenolate mofetil, 14 (18%) tacrolimus and 24 (31%) everlimus. Two patients received osteoporotic treatment: alendronate, n=1, and zoledronic acid, n=1.

**Table 1:** Main characteristics of the overall study population and patients with and without fracture after heart transplantation.

Variable	Study population n=79	No fracture n=58	Fracture n=21	P value
Age at transplantation (years)	54.1 ± 10.8	52.9 ± 10.9	57.6 ± 9.84	**0.04**
Male	61 (77%)	47 (81%)	14 (70%)	0.7
Weight (kg)	79.7 ± 16.0	81.2 ± 17.0	75.5 ± 12.4	0.43
BMI (kg/m2)	27.2 ± 4.99	27.6 ± 5.31	26.2 ± 3.91	0.34
Time from transplantation (months)	32.3 ± 35.0	33.3 ± 35.7	29.4 ± 33.8	0.46
Corticosteroids (mg/day)	10.0 ± 4.21	9.77 ± 4.28	10.8 ± 4.05	0.21
Diabetes mellitus	29 (37%)	21 (37%)	7 (33%)	0.77
Tobacco use	49 (62%)	38 (66%)	11 (52%)	0.29
Alcohol use	12 (15%)	9 (16%)	3 (14%)	1
Insufficient daily calcium intake	35 (58%)	27 (61%)	7 (47%)	0.32
Biological variables				
Calcium, mmol/l	2.26 ± 0.124	2.26 ± 0.121	2.26 ± 0.136	0.8
Phosphorus, mmol/l	1.08 ± 0.232	1.10 ± 0.238	1.02 ± 0.208	0.26
Creatinine, μmol/l	139 ± 67.9	139 ± 77.1	141 ± 32.1	0.082
eGFR, ml/min	51.0 ± 18.0	53.3 ± 18.6	44.8 ± 14.8	**0.011**
Parathyroid hormone, pg/ml	78.7 ± 68.5	82.0 ± 75.4	68.6 ± 40.3	0.6
25 hydroxy-vitamin D level, nmol/l	48.9 ± 26.4	48.2 ± 26.4	50.9 ± 27.1	0.73
>75	17 (23%)	13 (24%)	4 (19%)	
50-75	15 (20%)	10 (19%)	5 (24%)	1
<50	43 (57%)	31 (57%)	12 (57%)	0.95
B-ALP, μg/l	15.4 ± 8.87	15.3 ± 8.71	6.0 ± 9.67	
ßCTX, μg/l	0.379 ± 0.301	0.377 ± 0.298	0.384 ± 0.320	0.78
Osteocalcin, μg/l	21.2 ± 47.6	23.4 ± 54.2	14.0 ± 6.66	0.76

Data are mean ± SD unless indicated.

BMI, body mass index; eGFR, estimated glomerular filtration rate; B-ALP, bone-specific alkaline phosphatase; ßCTX, ß C-terminal collagen peptide. Bold values: significant result p (p<0.05).

### BMD and fracture assessment

**Figure 2:**
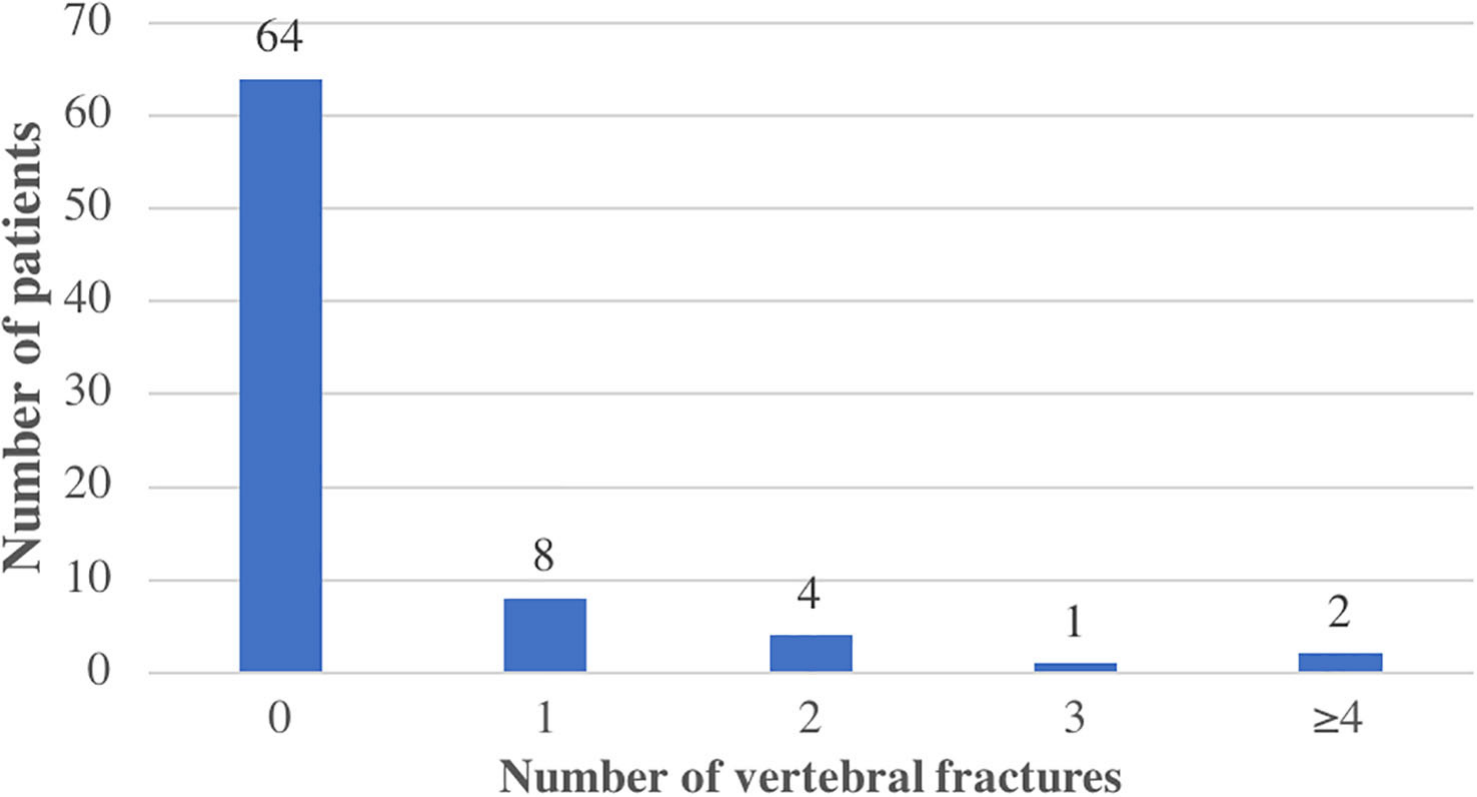
Vertebral fractures in the study population.

Osteopenia and osteoporosis were diagnosed in 38 (48%) and 22 (28%) patients, respectively. Diabetes mellitus was less prevalent in patients with than without osteoporosis: 4 (18%) versus 24 (44%), p=0.036 (**Table 2**). Pre-transplant osteoporotic fractures were noted for 2 patients (2 femoral neck fractures). At the time of the visit, 21 (27%) patients had fractures diagnosed after transplantation. Vertebral fractures were the most frequent (15, 71.4%). In total, 15 patients had 30 vertebral fractures (11 thoracic and 19 lumbar vertebral) (**Figure 2**). For 7 patients, vertebral fractures were unknown and were diagnosed on systematic X-rays. The other sites of fractures were femoral neck (n=2), pelvic (n=1), humeral (n=1), fibula (n=1), ad clavicular (n=1). Mean ages of patients with and without fracture after transplantation were 57.6±9.84 and 52.9±10.9 years, respectively (p=0.04). Estimated glomerular filtration rate (eGFR) was lower with than without fracture (44.8 ± 14.8 vs 53.3 ± 18.6, p=0.011), but osteoporosis was more frequent (9 [43%] vs 13 [22%], p=0.073) (**Table 3**). The prevalence of osteopenia did not significantly differ between the 2 groups. Mean BMD at the femoral neck and total hip was lower with than without fracture (femoral neck: 0.777 ± 0.125 vs 0.892 ± 0.174 g/cm², p<0.01; total hip: 0.892 ± 0.165 vs 0.748 ± 0.07 g/cm², p<0.001). The mean femoral neck and total hip T-scores were lower in patients with than without fractures: -1.79 ± 0.987 vs -0.955 ± 1.33 SD (p=0.01) and -2.07 ± 0.556 vs -0.976 ± 1.26 SD (p<0.001), but the difference was not significant for spine BMD. We performed a logistic regression analysis including age at transplantation eGFR and confirmed lower femoral neck BMD and total hip BMD for patients with fracture (OR 0.939 [95% CI 0.891–0.980], p<0.01, and OR 0.924 [95% CI 0.873–0.967], p<0.01, respectively). Age at transplantation and eGFR were not associated with fractures on multivariate analysis. The mean time from transplantation to the first fracture was 8.0 ± 7.6 months. Only two patients with fracture were < 50 years old at the time of transplantation (39 and 44 years) and 9 (43%) had a T-score <-2.5 SD at at least one site.

**Table 2:** Main characteristics of patients with and without osteoporosos after heart transplantation.

Variable	No osteoporosis n=55	Osteoporosis n=22	P value
Age at transplantation (years)	57.2 ± 10.5	55.1 ± 11.9	0.48
Male	45 (83%)	14 (64%)	0.075
Weight (kg)	81.8 ± 15.6	74.2 ± 16.8	0.075
BMI (kg/m2)	27.7 ± 5.08	26.0 ± 4.86	0.17
Time from transplantation (months)	32.5 ± 34.2	26.3 ± 29.0	0.42
Corticosteroids (mg/day)	9.66 ± 4.17	11.0 ± 4.46	0.24
Diabetes mellitus	24 (44%)	4 (18%)	**0.036**
Tobacco use	38 (69%)	12 (55%)	0.23
Alcohol use	10 (18%)	2 (9.1%)	0.49
Insufficient daily calcium intake	27 (60%)	8 (57%)	0.85
Biological variables			
Calcium, mmol/l	2.28 ± 0.124	2.23 ± 0.124	0.12
Phosphorus, mmol/l	1.08 ± 0.250	1.08 ± 0.190	0.96
Creatinine, μmol/l	130 ± 76.3	136 ± 39.2	0.48
eGFR, ml/min	52.4 ± 16.4	48.9 ± 21.7	0.5
Parathyroid hormone, pg/ml	70.8 ± 47.9	101 ± 105	0.19
25 hydroxy-vitamin D level, nmol/l	49.1 ± 26.7	49.3 ± 27.2	0.98
B-ALP, μg/l	14.4 ± 7.63	18.5 ± 11.3	0.16
ßCTX, μg/l	0.373 ± 0.329	0.408 ± 0.216	0.61
Osteocalcin, μg/l	14.8 ± 9.05	18.6 ± 11.1	0.2

Data are mean ± SD unless indicated.

BMI, body mass index; eGFR, estimated glomerular filtration rate; B-ALP, bone-specific alkaline phosphatase; ßCTX, ß C-terminal collagen peptide.Bold values: significant result p (p<0.05).

**Table 3:** Bone mineral density (BMD), T-score and prevalence of osteoporosis and osteopenia in the overall study population and in patients with and without fraction after heart transplantation.

Variable	Study population n=79	No fracture n=58	Fracture n=21	P value
BMD spine, g/cm^2^	1.04 ± 0.205	1.07 ± 0.169	0.937 ± 0.271	0.076
T-score spine, SD	-1.14 ± 1.44	-0.960 ± 1.40	-1.65 ± 1.48	0.14
BMD femoral neck, g/cm^2^	0.865 ± 0.170	0.892 ± 0.174	0.777 ± 0.125	**<0.01**
T-score femoral neck, SD	-1.16 ± 1.30	-0.955 ± 1.33	-1.79 ± 0.987	**0.01**
BMD total hip, g/cm^2^	0.859 ± 0.16	0.892 ± 0.165	0.748 ± 0.07	**<0.001**
T-score total hip, SD	-1.22 ± 1.22	-0.976 ± 1.26	-2.07 ± 0.556	**<0.001**
Osteopenia	38 (48%)	26 (45%)	12 (57%)	0.55
Osteoporosis	22 (28%)	13 (22%)	9 (43%)	**0.073**

Data are mean ± SD unless indicated.Bold values: significant result p (p<0.05).

### Biochemical variables

Biochemical variables are described in **Table 1**. At the time of the visit, 58 (77%) patients had vitamin D deficiency and 44% (n=34) had increased level of parathyroid hormone. Patients with and without fracture and with and without osteoporosis did not differ in 25 hydroxy-vitamin D level, percentage of vitamin D deficiency, or secondary hyperparathyroidism (p>0.05). Bone turnover was assessed with ßCTX, osteocalcin and B-ALP levels. Bone turnover did not differ between patients with and without fracture or those with and without osteoporosis. Only 9.7% of patients had an increased ßCTX level.

### Therapies

All patients received 2 or 3 immunosuppressant therapies. Overall, 90% (n=71) received calcineurin inhibitors and 86% (n=77) mycophenolate mofetil. Patients with and without fracture and with and without osteoporosis did not differ in immunosuppressant therapies. Dietary daily calcium intake was lower than recommended levels in 58% (n=35) of patients, with no difference between groups (with and without fracture or with and without osteoporosis).

### Therapeutic impact

After global bone fragility evaluation, calcium and/or vitamin D therapy was initiated in 58 (73.4%) patients. Specific osteoporosis treatment was started for 56 (70.9%): zoledronic acid (n=42), denosumab (n=8), alendronate (n=4), and teriparatide (n=2).

## Discussion

In this study, the prevalence of fractures was 27% in patients with heart transplantation since 2.6 years. We found a significant association between fractures and femoral neck and total hip BMD.

The prevalence of fractures in this work is similar to that observed in other studies. Leidig-Bruckner et al. found 21% of 105 patients with at least one vertebral fracture in the first year after heart transplantation and 27% at 2 years after (6). Another study reported 40% of fractures in 180 patients with heart transplantation less than 10 years’ previous (8). In our population, the mean time from transplantation to the first fracture was 8 months; this result confirmed a high risk during the first year after the transplantation. Previous studies showed a decrease in BMD during the first year post-transplantation (3–6). This observation could be explained by a high dose of corticosteroids to avoid acute graft rejection. Glucocorticoids reduce bone formation (16), induce apoptosis of osteoblasts and osteocytes (17), increase bone resorption and reduce intestinal calcium absorption (18). We did not find an association between prednisone dose and risk of fractures probably because of lack of data of cumulative corticosteroids dose. In our study, only 28% of patients were osteoporotic, and for patients with fracture, 43% had a T-score <-2.5 SD at at least one site. We found no association between spine BMD and risk of fracture, even though vertebral fractures were the most frequent in our population. BMD is an important factor for evaluating the risk of fracture but is insufficient to identify all patients at risk. Osteocyte apoptosis decreases bone quality, which results in early increased risk of fracture even before BMD decreases. The indirect effects of glucocorticoids could also be involved in the risk of fracture: reduced muscle mass leading to increased risk of falls (19).

We confirmed vitamin D deficiency in 57% of our patients, and 44% had secondary hyperparathyroidism. The vitamin D deficiency in heart transplant patients could be explained by heart disease, lack of exposure to sunlight and hepatic dysfunction. In candidates for heart transplantation, 17% had 25 hydroxy-vitamin D level < 9 ng/ml, with a significant association with bone turnover markers (20). The implication of vitamin D in bone metabolism and the high prevalence of severe deficiency in this population confirm that all transplantation patients and candidates should receive adequate supplementation. The objective is to maintain 25 hydroxy-vitamin D level > 75 nmol/l (30 ng/ml) (21).

We did not find a significant association between immunosuppressive therapies and fracture or osteoporotic state. The role of calcineurin inhibitors is controversial, with opposite results on bone turnover in rats treated with calcineurin inhibitors (22, 23). Carbonare et al. showed an association between femoral-neck T-score and daily cyclosporine dosage and suggested the protective effect of calcineurin inhibitors. In our work, the daily dosage of calcineurin inhibitors is unknown, and 90% of patients received calcineurin inhibitors, which could explain the lack of a significant difference (8).

Vertebral fractures are the most frequent localization of fractures in heart transplant patients. In our study, 15 patients had vertebral fractures, and for half of them the fractures were unknown and diagnosed on systematic radiography. This result remains that vertebral fracture could be asymptomatic. The International Society of Heart and Lung Transplantation proposed spine radiography for all adult heart transplant candidates (21). In case of acute dorsal or lumbar pain even without trauma in heart transplant patients or candidates, spine radiography (or CT if necessary) must be performed.

Our study has some limitations: the study was monocentric with a relative small number of patients. BMD was not assessed before heart transplantation, so the longitudinal evaluation of BMD after transplantation could not be evaluated. Data on cumulative glucocorticoids or calcineurin inhibitors use were not available. Nevertheless, the strength of our study was the systematic evaluation of BMD and radiography for all patients in a real-life study.

In summary, our study confirmed high vertebral fracture risk in heart transplant patients, especially during the first year after transplantation. The prevalence of vitamin D insufficiency and deficiency was high in this population. This study confirmed that more attention to bone fragility is necessary to reduce the fracture risk.

## Data availability statement

The raw data supporting the conclusions of this article will be made available by the authors, without undue reservation.

## Ethics statement

The studies involving humans were approved by The Institutional Review Board (No. 12-011) of Paris North Hospitals. The studies were conducted in accordance with the local legislation and institutional requirements. The participants provided their written informed consent to participate in this study.

## Author contributions

The authors confirm contribution to the paper as follows: study conception and design: MF, PhD. Data collection: MF, RC, CV, SO, EE, LD, EP, RD. Analysis and interpretation of results: MF, CV, RD, PhD. Draft manuscript preparation: MF, PhD. All authors contributed to the article and approved the submitted version.
